# Virtual Screening and Biological Evaluation of Inhibitors Targeting the XPA-ERCC1 Interaction

**DOI:** 10.1371/journal.pone.0051329

**Published:** 2012-12-14

**Authors:** Khaled H. Barakat, Lars P. Jordheim, Rolando Perez-Pineiro, David Wishart, Charles Dumontet, Jack A. Tuszynski

**Affiliations:** 1 Department of Physics, University of Alberta, Edmonton, Alberta, Canada; 2 Department of Engineering Mathematics and Physics, Fayoum University, Fayoum, Egypt; 3 Université de Lyon, Lyon, France; 4 INSERM U1052, Centre de Recherche en Cancérologie de Lyon, Lyon, France; 5 CNRS UMR 5286, Centre de Recherche en Cancérologie de Lyon, Lyon, France; 6 Department of Oncology, University of Alberta, Edmonton, Alberta, Canada; 7 Department of Biological Sciences, University of Alberta, Edmonton, Alberta, Canada; 8 Department of Computing Science, University of Alberta, Edmonton, Alberta, Canada; Southern Illinois University School of Medicine, United States of America

## Abstract

**Background:**

Nucleotide excision repair (NER) removes many types of DNA lesions including those induced by UV radiation and platinum-based therapy. Resistance to platinum-based therapy correlates with high expression of ERCC1, a major element of the NER machinery. The interaction between ERCC1 and XPA is essential for a successful NER function. Therefore, one way to regulate NER is by inhibiting the activity of ERCC1 and XPA.

**Methodology/Principal Findings:**

Here we continued our earlier efforts aimed at the identification and characterization of novel inhibitors of the ERCC1-XPA interaction. We used a refined virtual screening approach combined with a biochemical and biological evaluation of the compounds for their ability to interact with ERCC1 and to sensitize cells to UV radiation. Our findings reveal a new validated ERCC1-XPA inhibitor that significantly sensitized colon cancer cells to UV radiation indicating a strong inhibition of the ERCC1-XPA interaction.

**Conclusions:**

NER is a major factor in acquiring resistance to platinum-based therapy. Regulating the NER pathway has the potential of improving the efficacy of platinum treatments. One approach that we followed is to inhibit the essential interaction between the two NER elements, ERCC1 and XPA. Here, we performed virtual screening against the ERCC1-XPA interaction and identified novel inhibitors that block the XPA-ERCC1 binding. The identified inhibitors significantly sensitized colon cancer cells to UV radiation indicating a strong inhibition of the ERCC1-XPA interaction.

## Introduction

Nucleotide excision repair (NER) can be considered as an old friend, but is in fact a new enemy in the context of cancer. In normal cells, NER removes many types of DNA lesions, protecting cell integrity [1]. However, in cancer cells exposed to DNA damaging agents that distort the DNA helix or form bulky injuries to the genome, NER comes into play and removes the damage, thus protecting cancer cells from death [1], [2]. A striking example of this mechanism is represented by the use of platinum compounds such as cisplatin, the backbone for many treatments of solid tumors including testicular, bladder, ovarian, head and neck, cervical, lung and colorectal cancer [3]. It has been demonstrated that NER is the major DNA repair mechanism that removes cisplatin-induced DNA damage, and that resistance to platinum-based therapy correlates with high expression of ERCC1, a major element of the NER machinery [4], [5], [6], [7]. In this context, one way to increase the efficacy of platinum therapy and decrease drug resistance is to regulate NER by inhibiting the activity of ERCC1 and interacting proteins using novel therapeutic compounds [8].

The protein ERCC1 forms a heterodimer with XPF. The resulting complex is an endonuclease enzyme that cleaves the 5` end of the damage whereas XPG cleaves in the 3′ position (for a comprehensive review on NER, see ref. [2]). ERCC1-XPF is recruited to the damage site through a direct interaction between the centeral domain of ERCC1 and XPA, an indispensible element of the NER pathways [9], [10]. No cellular function beyond NER has been observed for XPA and competitive inhibition of the XPA interaction with peptide fragments is effective at disrupting NER [11], [12]. Furthermore, clinically, patients that have been shown to have low expression levels of either XPA or ERCC1 demonstrate higher sensitivity to cisplatin treatment, and people deficient for XPA (or other XP proteins) are hypersensitive to UV radiations [13], [14]. Hence, here we continue our earlier efforts aimed at the identification and characterization of novel inhibitors of the interaction between ERCC1 and XPA [15], in order to regulate the NER pathway and offer new alternatives to be added to the current NER and cell cycle inhibitor UCN-01(7-hydroxystaurosporine) [16]. The present work introduces a promising lead compound NERI01 (NER inhibitor 01) that targets the ERCC1-XPA interaction and sensitizes cancer cells to ultraviolet irradiation induced damage.

In the *in silico* part of our investigations, we employed a refined virtual screening protocol [17], [18] to screen the CNRS Chimiotheque Nationale (CN) library of investigative chemical compounds (∼50,000 structures) [19] against the binding site of XPA within 10 different ERCC1 models. The selected compounds were validated experimentally both after and before the exposure of cancer cells to UV radiation. One compound (termed here as NERI01) sensitized cells to UV radiation, strongly suggesting an activity through the regulation of the NER pathway, and was slightly synergistic with cisplatin in one cancer cell line. It is our hope that this newly discovered inhibitor would act as a template for the development of analogues that will improve the efficacy of platinum-based cancer therapy and ultimately lead to better cure rates.

## Results and Discussion

### Selection of an Initial ERCC1 Model

**Figure 1:**
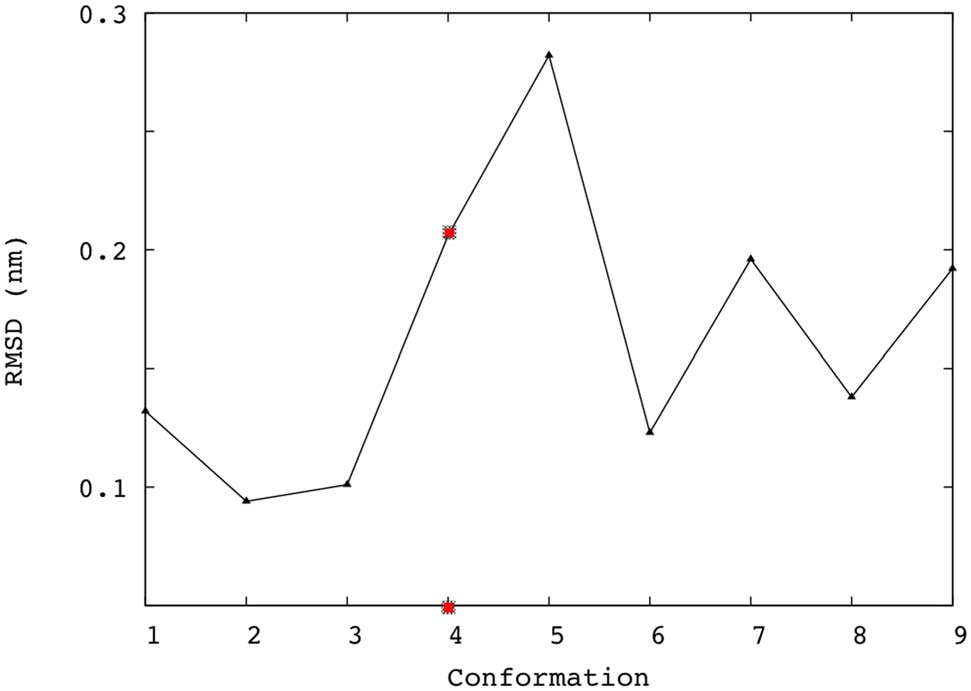
Selection of an initial ERCC1 target. The root mean square deviation (RMSD) of 9 ERCC1 NMR structures relative to an arbitrary NMR conformation. The centroid of the 9 structures (highlighted in red) was selected as the initial target structure against the full set of compounds included in the CN library.

It is always debatable whether to use target structures derived from MD simulations rather than from NMR data as a virtual model for protein structures. For example, Philippopoulos et al. suggested NMR structures as the most effective source for protein conformations [20]. A set of 15 NMR conformations for ribonuclease HI was compared to a trajectory obtained from a 1.7 ns MD simulation. The NMR data explored the conformational space of the protein more efficiently than the conventional MD simulation. In our present work, we exploited the published ERCC1-XPA NMR structure [12]. However, as the initial screening involved an enormous number of compounds (∼ 90,000) (see below), it was important to start the docking simulations using a representative target structure. This was done to reduce the computational cost without losing significant information related to the target flexibility. Focusing on the binding site, Figure 1 represents the RMSD of the relaxed 9 NMR conformations compared to the 10^th^ structure. We selected the centroid of the 9 structures to be our initial target. That is, we started the docking procedure using a binding site that has an equal RMSD separation from the other targets. The RMSD values ranged from 0.10 nm to 0.28 nm. Only two conformations were significantly separated from the reference conformation. Based on that, we choose conformation 4, with an RMSD separation of 0.21 nm to be our representative target. The rest of the structures were used in re-docking of the top 2,000 hits obtained from the initial single-target screening (see below).

### A Two-phase Docking Protocol

Once we had selected a representative conformation from the NMR ensemble, we carried out a two-stage docking simulation. The first stage was a preliminary search for potential binders to ERCC1 using the full set of the CN library. This search resulted in a wide spectrum of the binding energy values ranging from −11 kcal/mol to 20 kcal/mol. Based on our experience and on similar studies in the literature, we decided to truncate the hit list at −5 kcal/mol. Taking the population of the largest cluster to be greater than 25%, this energy cutoff resulted in a set of 2,000 hits ranked according to AutoDock scoring function.

The second stage was a more rigorous docking approach that employed the RCS methodology [21]. In the RCS approach, all-atom MD simulations (e.g., 2–5 ns simulation) are applied to explore the conformational space of the target, while docking is subsequently used for the fast screening of drug libraries against an ensemble of receptor conformations. This ensemble is extracted at predetermined time intervals (e.g., 10 ps) from the simulation, resulting in hundreds of thousands of protein conformations. Each conformation is then used as a target for an independent docking experiment. The RCS methodology has been successfully applied to a number of cases. An excellent example is that of an HIV inhibitor, raltegravir which became the first FDA approved drug targeting HIV integrase [22], [23]. Other successful examples include the identification of novel inhibitors of the acetylcholine binding protein [24], RNA-editing ligase 1 [25], the influenza protein neuraminidase [26] and *Trypanosoma brucei* uridine diphosphate galactose 4′-epimerase [27]. These applications employed alternative ways to solve two main problems with the method, namely, reducing the number of extracted target conformations and deciding on how to select the final set of hits after carrying out the screening process. For the first problem, a number of studies suggested extracting the structures at larger intervals of the MD simulation (e.g. every 5 ns or so), [24] condensing the structural ensemble generated from MD simulations using QR factorization, [25] or clustering the MD trajectory using root-mean-square-deviation (RMSD) conformational clustering, [26], [27] On the other hand, to rank the screened compounds and suggest a final set of top hits, some studies used only docking predictions, [24], [25], [26]while others suggested (as in this thesis) using a more accurate scoring method (e.g. MM/PBSA (Molecular Mechanics/Poisson Boltzmann Surface Area)) to refine the final selected hits. [21] All of these approaches, similar to the work presented here, were aiming at keeping the balance between significantly reducing the number of target structures and, in the meantime, retaining their capacity to describe the conformational space of the target.

**Figure 2:**
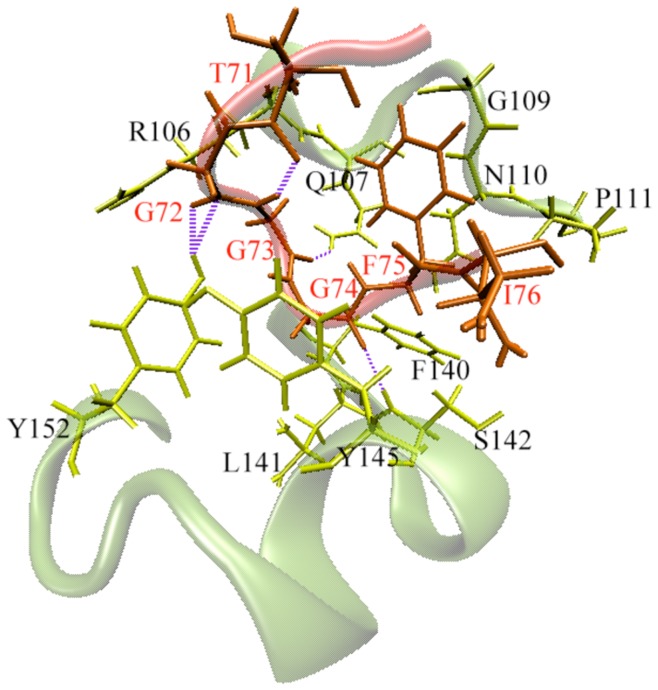
ERCC1-XPA interactions. The binding between ERCC1 (teal) and XPA (red) is primarily mediated by 5 residues from XPA peptide, namely; G72, G73, G74, F75 and I76. On the other hand, the contribution from the ERCC1 binding site is distributed among 10 residues; R106, Q107, G109, N110, P111, F140, L141, S142, Y145 and Y152.

To partially introduce receptor flexibility within the docking, the top 2,000 hits from the initial screening were re-docked against the remaining 9 NMR conformations. As expected, this produced a new ranking for the 2,000 hits. At this stage, autodock-scoring function and an adaptive clustering method (see methodology) were used to suggest a preliminary ranking of the 2,000 compounds. After that, visual inspection combined with this scoring method reduced the 2,000 hits to only 200 molecules that have acceptable population size (see below). We noticed that most of them are properly fitted within the ERCC1 pocket. The binding energies of the successfully docked structures (∼ 170 hits) ranged from −12 kcal/mol to −7 kcal/mol. It is worth mentioning that the binding site of ERCC1 has limited flexibility. Based on our previous investigations, [15], the important residues that mostly contribute to its interaction with ligands are Gly109, Pro111, Asn110, Asp 129, Phe140, Tyr145, and Arg156 (Figure 2). However, most of the binding energy values obtained from the two docking stages were not statistically significant. The separation between the energies was not able to select hits for experimental testing based on docking results. Therefore, we decided to perform MD simulations on the top 170 RCS hits starting from their minimal energy conformations within the ERCC1 binding site.

### Clustering of Docked Conformations and Extraction of Binding Modes

Docking simulations produce massive numbers of possible solutions. Each proposed solution represents a potential binding mode for the tested ligand within the targeted site. Mining these data sets and pulling out the most probable solution for each compound is tricky and requires careful treatment. We developed an iterative clustering algorithm that takes into account a couple of clustering metrics (see Materials and Methods). This adaptive approach was tested on other targets and led to successful outcomes [28], [29], [30]. For MD simulations, starting from the optimal binding mode is the most efficient route to reach equilibrium. Therefore, by running the clustering protocol on each ligand and filtering the hits in terms of the population of the largest cluster (see Materials and Methods), we were able to prepare a set of 170 distinct hits ranked by their binding energies. The selected hits were subjected to all-atoms, explicit solvent MD simulations.

### MD Simulations on Promising Hits

MD simulations introduced target flexibility to the molecular recognition problem. It allowed all protein side chains to move, rotate and interact with the different parts of the ligands. The conclusion reached after running MD simulations on the complexes was decisive and provided answers to many relevant inquiries, in particular: “Was the binding mode stable and realistic? How did the ligand stability evolve in time? What were the major interactions that made this ligand bind? Were there any water-mediated interactions involved?”.

**Figure 3:**
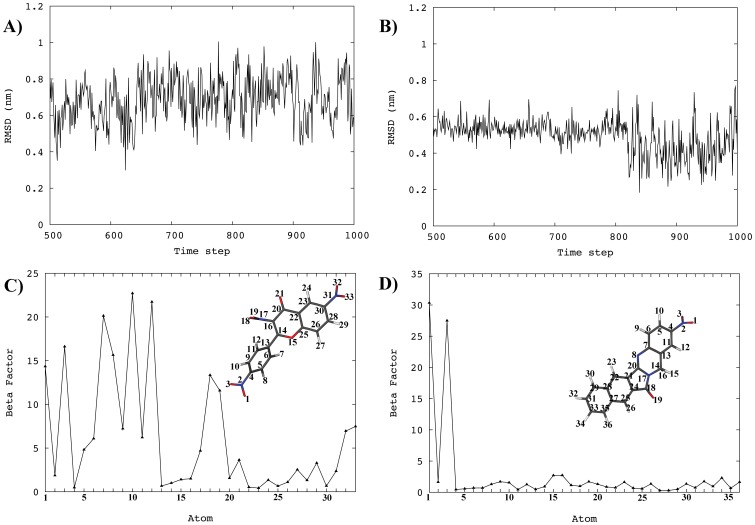
Stability of two selected hits. RMSD of NERI01 (A) and of AB-00031382 (B). Atomic fluctuations of NER01 (C) and of AB-00031382 (D). The two molecules are also shown with atom numbers as a reference for their atomic fluctuations. See text for details.

**Figure 4:**
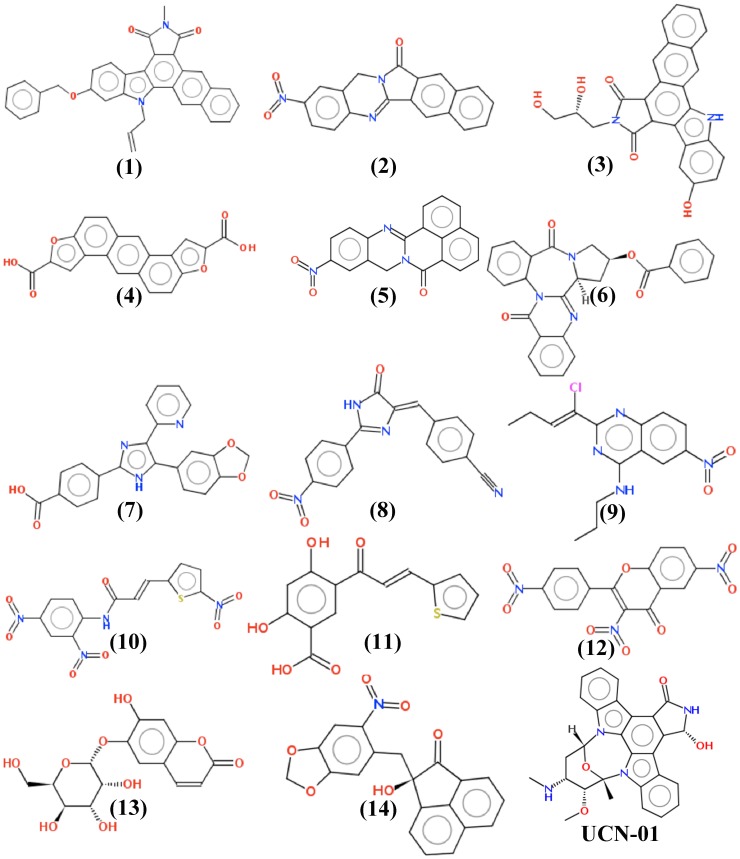
Structures of the 14 experimentally tested compounds. NERI01 is compound 12.

Approximately half of the docking-predicted hits were stable within the binding site. They had proper interactions with various regions of the target. They also formed hydrogen bonds directly with the protein side chains or indirectly through water molecules. As an example, Figure 3 shows the RMSD and atomic fluctuations of two selected hits; NERI01 (compound 12 in Figure 4 and Table 1, also known as AB-00026258) and a similar lead structure (compound 2 in Figure 4 and Table 1). The average RMSD for the two compounds was around 6 Å, which is consistent with values obtained in similar studies [31]. The RMSD for NERI01 (Figure 3-A) was more fluctuating than that of the other compound (Figure 3-B), indicating higher flexibility. This was evident in the atomic fluctuatation analysis. Many parts of NERI01 are flexible (Figure 3-C) including the three nitro groups and the single rotatable bond in the middle of its structure. On the other hand, the other compound is mainly rigid (Figure 3-D) with only partial flexibility in the nitro group. The flexibility of NERI01 seems to play an important role in establishing many hydrogen bonds within the ERCC1.

**Table 1:** Ranking of the selected hits using the MM-PBSA method compared to that of AutoDock.

#	MM-PBSA Rank	AutoDockRank	MM-PBSA BE (kcal/mol) ±0.5	LogP
1	1	185	−11.3	4.7
2	2	62	−11.0	3.4
3	5	139	−10.7	3.1
4	9	112	−9.9	4.4
5	4	63	−9.6	3.7
6	11	128	−9.3	3.3
7	13	59	−8.9	3.4
8	23	181	−8.2	2.7
9	29	138	−7.7	4.3
10	34	54	−7.4	2.6
11	37	131	−7.2	3.1
12	45	104	−7.0	2.5
13	46	44	−6.9	−0.1
14	47	129	−6.8	3.4

**Figure 5:**
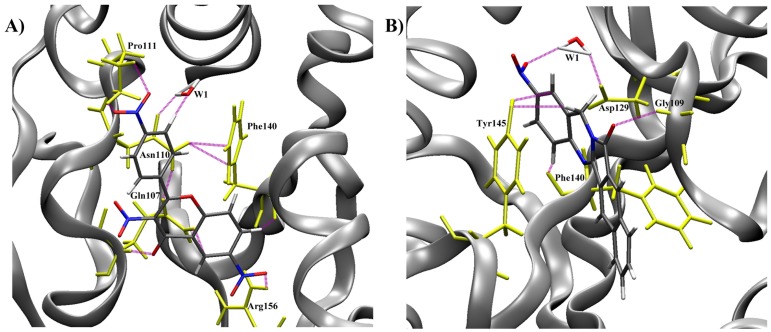
Binding modes and hydrogen bonding of the two selected hits shown in Figure 3. Binding mode of NERI01 (A) and of AB-00031382 (B).

Figure 5 illustrates the binding mode of the two compounds and shows their hydrogen bond network within the binding site. NERI01 (Figure 5-A) made 6 hydrogen bonds with ERCC1. The oxygen of the first nitro group was hydrogen-bonded to the side chain of Pro111. A water molecule (W1) mediated a hydrogen bond between the ligand and the side chain of Asn110. One more hydrogen bond connected the middle of NERI01 to the backbone of Gln107, while another hydrogen bond connected the other side of the compound to the backbone of Phe140. The last hydrogen bond attached the other nitro group to the side chain of Arg156. Noticeably, NERI01 stabilized the interaction between the side chains of Phe140 and Asn110 allowing them to build two hydrogen bonds, bringing them close enough to provide a hydrophobic cleft to the aromatic regions of NERI01. For compound 2 (Figure 5-B), although a similar binding mode was observed, fewer hydrogen bonds existed. A water molecule (W1) mediated a hydrogen bond between the nitro group and the side chain of Asp129. Two hydrogen bonds connected the ligand to the backbones of Phe140 and Gly109, respectively. Tyr145 was hydrogen-bonded to the middle of the compound. Finally, the large hydrophobic region of the compound interacted with the side chain of Phe140.

Thus, after the detailed analysis of the binding modes for most of the top hits, common binding motifs can be observed. First, one to two hydrogen bonds existed between the ligands and Pro111 or Tyr145, with a rigid moiety occupying the hydrophobic region between Phe140 and Tyr145. Second, a water molecule can mediate a hydrogen bond between the ligands and Asn110 or Asp129. Finally, Arg156 can provide a hydrogen bond to a polar moiety of the ligand bringing it closer to the hydrophobic region of Phe140. Observing these general features is essential in order to further optimize the compounds and achieve a greater affinity for the target.

### Binding Energy Analysis and Rescoring

Besides using MD simulations to refine the docked structures, another essential constraint for a successful VS experiment is to accurately predict their binding energies. To correctly fulfill this task, we moved far from the simple AutoDock scoring function (Eq. 1). However, we were also restricted by the need to have a reasonably fast method that can be applied to many systems. At this stage, it was also necessary to consider various factors that were either ignored or neglected during the initial docking scoring, such as solvation and entropic terms. In this context, our VS protocol utilized the MM-PBSA [32] to suggest the final ranked set of top hits (see Materials and Methods). The method combines molecular mechanics with continuum solvation models. It has been extensively tested on many systems and shown to reproduce, with an acceptable range of accuracy, experimental binding data. It was also validated as a VS refining tool and revealed excellent results in predicting the actual binding affinities and in discriminating true binders from inactive (decoy) compounds [33], [34], [35]. Its main advantages are the lack of adjustable parameters and the option of using a single MD simulation for the complete system to determine all energy values.

Table 1 compares the MM-PBSA ranking to that of AutoDock for the 14 compounds that were retained for biological evaluation. Only these compounds showed acceptable solubility as predicted by the software ADMET predictor. The ranking of AutoDock is clearly different from that of MM-PBSA. For example the top MM-PBSA-hit (compound 1) was ranked as 185 using AutoDock scoring, while NERI01 was ranked as 104. This huge difference in ranking between the two methods undoubtedly states the weakness of AutoDock scoring in filtering true binders from false positives. Figure 4 shows the structure of the 14 tested hits. NERI01 has a less bulky structure than most of the compounds. A very similar structure to NERI01 is compound 12, which also has a slightly better scoring according to MM-PBSA (see Table 1). The nitro group is obvious in most of the compounds with alternatives of polar substituents for the rest of the structures. The higher the hydrophobicity of the compound is, the better its binding energy to the ERCC1 binding site.

### Validation of Binding Affinity Through the Binding Kinetics Assays for Selected Ligands

In order to confirm the binding affinity for the target protein of the top hit compounds we have undertaken to perform direct measurements of the interaction between compounds 10 and 12 and a peptide that contains the binding domain of ERCC1 with XPA, ERCC1^92–214^. ERCC1^92–214^ corresponds to 123 amino-acids of ERCC1 containing the interacting domain with XPA. Its concentration was 2 mg/ml. The peptide AF-41 corresponds to 41 amino-acids of XPA containing the interacting domain with ERCC1. Its concentration is 1.2 mg/ml. The two peptides were synthetic and obtained with a purity of approximately 85% from Proteogenix (Oberhausbergen, France). They were both diluted in HBS-EP buffer (0.01 M HEPES pH 7.4, 0.15 M NaCl, 3 mM EDTA, 0.005% surfactant P20). The amino acid sequences for the two peptides, their purity and molecular weights were determined using mass spectroscopy and HPLC techniques and the relevant reports are available in the Supplementary Information material.

**Figure 6:**
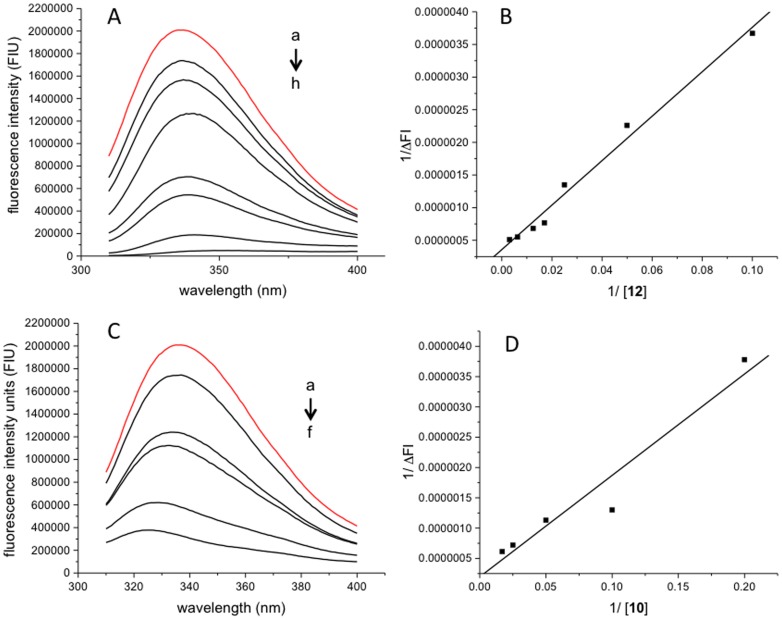
Fluorescence intensity profiles (A and C) and the corresponding plots of 1/ΔFI versus [L] (B and D) for ERCC1^92–214^ in presence of compounds 12 (A and B) and 10 (C and D). Fluorescence intensity profiles were obtained by monitoring the Tryptophan quenching of ERCC192-214 (20 µM) in the presence of ligand 12 (a-0 µM, b-10 µM, c-20 µM, d-40 µM, e-60 µM, f-80 µM, g-160, h-320 µM) and 10 (a-0 µM, b-5 µM, c-10 µM, d-20 µM, e-40 µM, f-60 µM) at the excitation wavelenght of 295 nm. In red, fluorescence intensity profile for ERCC1^92–214^ alone.

**Figure 7:**
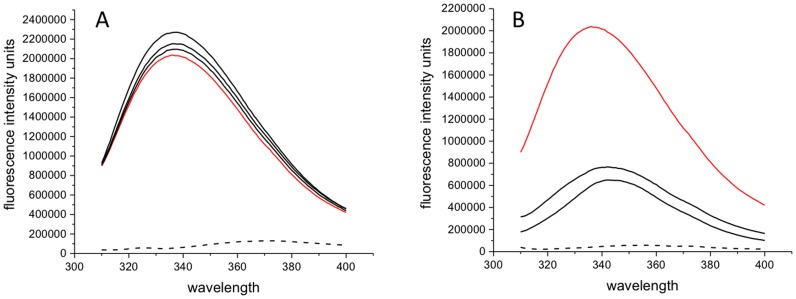
Fluorescence quenching intensity profiles of ERCC1^92–214^ (20 µM, red line) in the presence of caffeine (0 µM dash line, 40 µM, 80 µM and 320 µM black line) in HBS-EP buffer (0.01 M HEPES pH 7.4, 0.15 M NaCl, 3 mM EDTA, 0.005% surfactant P20). λexcit-295 nm, slit width 4 nm.

Experimental evidence of the binding of ligands **10** and **12** to ERCC1^92–214^ peptide was obtained by using fluorescence experiments. When excited at 295 nm ERCC1^92–214^ exhibits an intrinsic fluorescence due to the presence of two tryptophans residues in the polypeptide chain which is notably quenched upon addition of incremental concentrations of the ligands, as a result of a binding event (Figure 6A and C). The binding constant values were estimated to be 3.7±0.1×10^4^ M^−1^ and 1.5±0.1×10^4^ M^−1^ for compounds **10** and **12,** respectively, and the dissociation constant (*K*_d_) calculated to be 27.4 µM and 66.8 µM respectively (Figure 6B and D). In Fig. 7 we have illustrated the lack of fluorescence quenching response for a negative control chosen to be caffeine in solution consisting of the HBS-EP buffer (0.01 M HEPES pH 7.4, 0.15 M NaCl, 3 mM EDTA, 0.005% surfactant P20) and with the same peptide ERCC1^92–214^.

We believe that the data collected from fluorescence quenching experiments should not be significantly affected by the presence of ligand aggregation. In fact, according to the pertinent literature, this fluorescence technique has been very useful to discriminate between specific and nonspecific inhibition [36]. Ligand aggregation is more prompt to induce the presence of false positives in enzymatic assays where, once formed, they can sequester proteins and non-specifically inhibit their activity and also in SPR analysis where the accumulation of material onto the microchip surface interferes with the measurement. Another piece of evidence that supports the presence of specific interactions between ERCC1^92–214^ and the ligands is provided by the calculation of the biomolecular quenching rate constant K_Q_ for compounds **12** (1.50×10^12^ Ms^−1^) and **10** (3.66×1012 Ms^−1^) through the following equation: K_A_ = K_Q_ τ_0_ [37], where K_A_ is the association constant, K_Q_ is the biomolecular rate quenching rate constant and τ_0_ is the average lifetime of the biomolecule without a quencher (τ_0_ = 10^−8^ s) [38]. The results obtained from this study show that the estimated values for K_A_ are greater than the maximum scatter quenching constant of various quenchers with the biopolymers (K_Q_ = 2×10^10^ Ms^−1^) [39] which indicates that the observed static quenching for both ligands is caused by the formation of a non-fluorescent ground state fluorophore-quencher complex. Based on these facts, the presence of large aggregates would most likely interfere with the complex formation due to steric effects therefore cancelling the quenching effect [36], contrary to what is observed experimentally. Additionally, all the experiments were performed in the presence of P20 and we think that it reduces considerably the chances of having aggregate compounds in the mixture.

### NERI01 Sensitize Cells to UVC Irradiation

As aforementioned, NER is a major DNA repair pathway that eliminates DNA lesions induced by UV light [40]. A deficiency in NER leads to dramatic diseases characterized by hypersensitivity to UV and a prominent clinical and genetic heterogeneity. Among the diseases provoked by inactive NER pathway is the Xeroderma Pigmentosum (XP) disease. XP is a direct consequence of lacking one out of several NER proteins such as XPA [41]–[43]. A major syndrome of XP is the hypersensitivity to UV radiation and, consequently, a high susceptibility to produce skin cancer. As the role of XPA within the NER mechanism is to interact with ERCC1 and recruit the XPF-ERCC1 endonuclease to the damage, we thought that a straightforward and sufficient filter of compounds that target the ERCC1-XPA interaction is to test their ability to sensitize cells to UV radiation. The more UV sensitization induced, the stronger the compound in targeting this interaction.

**Figure 8:**
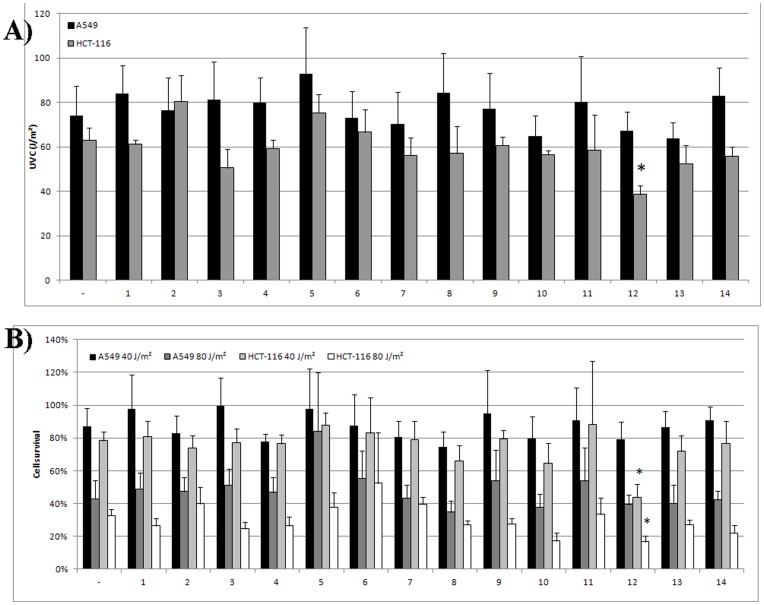
Sensitivity of cancer cells to UVC irradiation alone or in combination with potential inhibitors of the interaction between ERCC1 and XPA. IC50 values (J/m^2^) (A) and cell survival (B) were determined as indicated in material and methods. ^*^: p<0.05 as compared to cells without inhibitor using Student’s *t*-test.

The selected 14 molecules were evaluated for their potential to sensitize human colon (HCT-116) and lung (A549) cancer cells to UVC irradiation. Figure 8 describes the effects of the compounds on the tested cell lines. Most of the compounds showed little activity in sensitizing cells to UVC radiation. The most significant effect was of AB-00026258 (termed as NERI01), in particular for HCT-116 cells, with a decrease in the IC50 and the percentage of survival. Indeed, the IC50 values decreased from 63.0 J/m^2^ to 38.7 J/m^2^ in HCT-116 cells incubated in absence and in presence of the inhibitor respectively. Moreover, cell survival after exposition to 40 or 80 J/m^2^ decreased from 78.3% to 43.8% and from 32.8% to 16.8% respectively. These results are in agreement with the previous data indicating approximately 2-fold decrease in both UVC and cisplatin IC50 in cells with siRNA induced decrease in XPA (Nagao A 2008 BBRC, Cummings M 2006).

Compound 12 was assessed for synergy with cisplatin in two cancer cell lines. Combination indexes 95 (CI_95_) were 0.80 and 0.97 in HCT116 and A549 cells indicating slight synergy and additivity respectively (Table 2).

**Table 2:** Inhibitory concentrations 50 (IC_50_) and CI95 for cisplatin and compound 12 in HCT116 and A549 cells.

	Cisplatin IC_50_ (µM)	12 IC_50_ (µM)	CI_95_
HCT116	5.34±1.94	4.80±1.80	0.80±0.22
A549	5.41±1.75	17.50±5.25	0.97±0.09

Results are mean values from three independent experiments ± standard error of means. Synergy is defined as CI_95_<0.9, additivity for 0.9< CI_95_<1.1 and antagonism as CI_95_>1.1.

AB-00027849 (compound 10 in Table 1 and Figure 4) has almost the same structure of NERI01. The compound comprises the three-nitro groups, however, it is less bulky and more flexible than NERI01. Although the observed effect of AB-00027849 is less significant than of NERI01 (Figure 8), the detected biological activity reveals an importance to the general scaffold presented in the two structures. In other words, NERI01 can be used as a starting point for inhibitors of the ERCC1-XPA interaction.

## Materials and Methods

### Ligand Preparation

The CN database originally includes ∼50,000 compounds. One problem we found in using these structures is that they are represented by 2D SDF-format with no hydrogen atoms attached. This required a number of cleaning and preparation steps before we were able to use them in our VS simulations. For this purpose, we employed the software LigPrep from the Schrödinger package [44] to translate the 2D information into its 3D representative structure. LigPrep also generated variants of the same ligand with different tautomeric, stereochemical, and ionization properties. The final set of compounds constituted approximately 90,000 chemical structures. All generated structures were conformationally relaxed using energy minimization protocols included in LigPrep.

### Target Preparation

Our next step relied on Tsodikov’s NMR crystal structure of XPA bound to ERCC1 (PDB entry 2JNW) [12]. The NMR ensemble included 10 different conformations for the proteins; all of them were used in this study. The binding site was characterized in our previous work (see Figure 2) [15]. In this model, the central domain of ERCC1 (residues 99–214) is bound to a fragment of XPA (residues 67–77). Prior to docking, the XPA peptide was removed, protonation states of the residues constituting the ERCC1 pocket were adjusted using the software PDB2PQR [45], and the protein structures were conformationally relaxed using the NAMD molecular dynamics software with constraints on the backbone atoms (see below).

### Docking Protocol

All docking simulations employed the software AutoDock, version 4.0 [46]. The docking method and parameters were similar to the ones used in our previous work [15]. The screening method adopted two filtering phases with the same docking parameters. First, we screened the entire CN library against a single target model followed by applying the relaxed complex scheme (RCS) [47] through docking of the top 2,000 hits from the first screen against the rest of the ten target structures (see results for more details). Using the Lamarckian Genetic Algorithm (LGA), the docking parameters included an initial population of 150 random individuals; a maximum number of 10,000,000 energy evaluations; 100 trials; 27,000 maximum generations; a mutation rate of 0.02; a crossover rate of 0.80 and the requirement that only one individual can survive into the next generation.

### Clustering and Preliminary Ranking of Hits

Clustering of the docking results followed the same adaptive procedure as the one employed in our previous study [15]. In brief, for each docking simulation a modified version of the PTRAJ module of AMBER [48] clustered the docking trials. Every time a number of clusters were produced, two clustering metrics (i.e. DBI and percentage of variance [49]) were calculated to assess the quality of clustering. Once acceptable values for these metrics were reached, the clustering protocol extracted the clusters at the predicted cluster counts. The screening protocol then sorted the docking results by the lowest binding energy of the most populated cluster. If more than one target was involved, as it was the case for the second phase of docking (see above), a different ranking scheme was followed. The objective was to extract the docking solution, for each ligand, that had the largest cluster population and the lowest binding energy from all targets. In this context, for each ligand, the docking results were clustered independently for the individual targets. The clustering results were then compared and only the ones that corresponded to at least 25% as a cluster population were considered. AutoDock scoring function (Eq. 1) provided a preliminary ranking for the compounds.
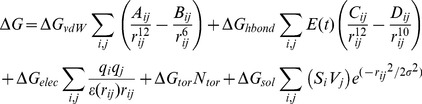
(1)


Here, the five 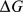 terms on the right-hand side are constants. The function includes three in vacuo interaction terms, namely a Lennard-Jones 12-6 dispersion/repulsion term, a directional 12-10 hydrogen bonding term, where E(*t*) is a directional weight based on the angle, *t*, between the probe and the target atom, and screened Columbic electrostatic potential. In addition, the unfavorable entropy contributions are proportional to the number of rotatable bonds in the ligand and solvation effects are represented by a pairwise volume-based term that is calculated by summing up, for all ligand atoms, the fragmental volumes of their surrounding protein atoms weighted by an exponential function and then multiplied by the atomic solvation parameter of the ligand atom (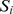).

Thus, the binding energies of the selected clusters were sorted and only the cluster with the lowest energy was retained for further analysis. Following this procedure, we selected 200 hits for more rigorous Molecular Dynamics (MD) simulations and binding energy analysis.

### Molecular Dynamics Simulation

The lowest energy pose for each ligand with its representative ERCC1 structure was used as a starting configuration of an MD simulation. The AMBER99SB force field [50] was used for protein parameterization, while the generalized AMBER force field (GAFF) provided parameters for ligands [51]. For each ligand, partial charges were calculated with the AM1-BCC method using the Antechamber module of AMBER 10. Protonation states of all ionizable residues were calculated using the program PDB2PQR. All simulations were performed at 300 K and pH 7 using the NAMD program [52]. Following parameterization, the protein-ligand complexes were immersed in the center of a cube of TIP3P water molecules. The cube dimensions were chosen to provide at least a 15 Å buffer of water molecules around each system. When required, chloride or sodium counter-ions were added to neutralize the total charge of the complex by replacing water molecules having the highest electrostatic energies on their oxygen atoms. The fully solvated systems were then minimized and subsequently heated to the simulation temperature with heavy restraints placed on all backbone atoms. Following heating, the systems were equilibrated using periodic boundary conditions for 100 ps and energy restraints reduced to zero in successive steps of the MD simulation. The simulations were then continued for 2 ns during which atomic coordinates were saved to the trajectory every 2 ps for subsequent binding energy analysis.

### Binding Free Energy Calculation and Rescoring of Top Hits

This study utilized the molecular mechanics Poisson-Boltzmann surface area (MM-PBSA) technique to rescore the preliminary ranked docking hits [32]. It combines molecular mechanics with continuum solvation models. The total free energy is estimated as the sum of average molecular mechanical gas-phase energies (E_MM_), solvation free energies (G_solv_), and entropy contributions (-TS_solute_) of the binding reaction:
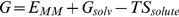
(2)


The molecular mechanical (*E*_MM_) energy of each snapshot was calculated using the SANDER module of AMBER10 with all pair-wise interactions included using a dielectric constant (ε) of 1.0. The solvation free energy (*G*_solv_) was estimated as the sum of electrostatic solvation free energy, calculated by the finite-difference solution of the Poisson–Boltzmann equation in the Adaptive Poisson-Boltzmann Solver (APBS) and non-polar solvation free energy, calculated from the solvent-accessible surface area (SASA) algorithm. The solute entropy was approximated using the normal mode analysis. Applying the thermodynamic cycle for each protein-ligand complex, the binding free energy was approximated by:
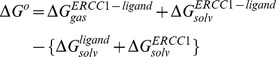
(3)


Here, (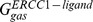) represents the free energy per mole for the non-covalent association of the ligand-protein complex in vacuum (gas phase) at a representative temperature, while (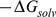) stands for the work required to transfer a molecule from its solution conformation to the same conformation in vacuum (assuming that the binding conformation of the ligand-protein complex is the same in solution and in vacuum).

### Partition Coefficient (LogP) Solubility Analysis

Top ranked structures were exported to the software ADMET Predictor (Simulations Plus) to estimate their solubility and log P values [53].

### Source of Compounds

Fourteen of the top compounds were obtained through Chimiothèque National-collaborating laboratories. Most compounds have not been reported elsewhere, but the synthesis of AB-00005094 [54], AB-00012818 and AB-00012800 [55], [56] have previously been published.

### Fluorescence Quenching Measurements of Binding Kinetics

Fluorescence measurements were made on a PTI MODEL-MP1 spectrofluorometer using a 10 mm path length cell. The excitation wavelength of 295 nm was used, and the scan range was 310–450 nm. Excitation and emission slit widths of 4 nm were used. Steady state fluorescence of the LA-123 peptide in HBS-EP buffer (0.01 M HEPES pH 7.4, 0.15 M NaCl, 3 mM EDTA, 0.005% surfactant P20) was measured by fixing the peptide concentration at 20 µM and adding aliquots of ligands **10** and **12** (10 mM in DMSO stock solutions) in the concentration range 0–320 µM. Data from the fluorescence quenching experiments were used to determine the apparent binding constant of the ERCC1^92–214^ peptide in the presence of the ligands according to
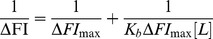
(4)where 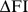 is the change in the peptide fluorescence in the presence of the ligands, 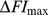 is the maximal change in fluorescence intensity, *K*_b_ is the binding constant and [L] is the concentration of ligand added. From the slope of the linear plot of 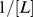 versus 1/ΔFI, the binding constant (*K*_b_) and dissociation constant (*K*_d_ = 1/*K*_b_) were estimated. The results were expressed as mean values ± SD (n = 5−7). The inner filter effects were corrected empirically by measuring the change of fluorescence intensity of a tryptophan solution equivalent to the ERCC1^92–214^ peptide concentration in the presence of the ligands, and the corrected fluorescence intensities were used for all calculations.

### Ultraviolet (UV) Cell Survival Assay

A549 and HCT116 cell lines we used were obtained from the ATCC Cell Biology Collection. Cells (A549 or HCT116 cell lines) were seeded in 12-well or 24-well plates with 50,000 or 20,000 cells per well in a final volume of 750 or 400 µl of DMEM media (Invitrogen, Cergy Pontoise, France) containing L-glutamine, penicillin (200 IU/ml), streptomycin (200 µg/ml) and 10% fetal bovine serum (Invitrogen, Cergy Pontoise, France), and incubated overnight at 37°C in presence of 5% CO_2_. Media was removed and cells were washed twice with PBS 1X and exposed to different doses of UVC irradiation in a Spectrolinker XL 1000 (Spectronics Corporation). Media with or without potential inhibitors was added and cells were incubated for another 72 hours before living cells were quantified with methylthiazoletetrazolium (MTT) assay as previously described [57].

### Evaluation of Synergy Effects

Cells (3,000 per well) were seeded in 100 µl cell culture media in 96-well plates and adhered for 24 hours before different concentrations of cisplatin alone, compound 12 or a mixture of a fixed concentration ratio of the two compounds were added. After incubation for another 72 hours before living cells were quantified with the MTT assay. Values for inhibitory concentrations 50 (IC_50_) and combination index 95 (CI_95_) were calculated with CompuSyn software 1.0 (ComboSyn, Inc., USA), and results expressed as mean of three independent experiments.
